# Gray values and noise behavior of cone-beam computed tomography machines—an *in vitro* study

**DOI:** 10.1093/dmfr/twae053

**Published:** 2024-11-19

**Authors:** Nicolly Oliveira-Santos, Hugo Gaêta-Araujo, Rubens Spin-Neto, Dorothea Dagassan-Berndt, Michael M Bornstein, Matheus L Oliveira, Francisco Haiter-Neto, Deborah Q Freitas, Ralf Schulze

**Affiliations:** Department of Oral Surgery and Stomatology, Division of Diagnostic Sciences, School of Dental Medicine, University of Bern, Bern, 3010, Switzerland; Graduate School for Health Sciences, University of Bern, Bern, 3012, Switzerland; Department of Stomatology, Public Health and Forensic Dentistry, Division of Oral Radiology, Ribeirao Preto School of Dentistry, University of Sao Paulo, Ribeirao Preto, 14040-904, Brazil; Department of Dentistry and Oral Health, Section for Oral Radiology and Endodontics, Aarhus University, Aarhus, 8000, Denmark; Center for Dental Imaging, University Center for Dental Medicine Basel UZB, University of Basel, Basel, 4058, Switzerland; Department of Oral Health and Medicine, University Center for Dental Medicine Basel UZB, University of Basel, Basel, 4058, Switzerland; Department of Oral Diagnosis, Piracicaba Dental School, University of Campinas, Piracicaba, 13414-903, Brazil; Department of Oral Diagnosis, Piracicaba Dental School, University of Campinas, Piracicaba, 13414-903, Brazil; Department of Oral Diagnosis, Piracicaba Dental School, University of Campinas, Piracicaba, 13414-903, Brazil; Department of Oral Surgery and Stomatology, Division of Diagnostic Sciences, School of Dental Medicine, University of Bern, Bern, 3010, Switzerland; Graduate School for Health Sciences, University of Bern, Bern, 3012, Switzerland

**Keywords:** cone-beam computed tomography, CBCT, gray values, phantoms, imaging

## Abstract

**Objectives:**

To systematically evaluate the mean gray values (MGVs) and noise provided by bone and soft tissue equivalent materials and air imaged with varied acquisition parameters in 9 cone-beam computed tomography (CBCT) machines.

**Methods:**

The DIN6868-161 phantom, composed of bone and soft tissue equivalent material and air gap, was scanned in 9 CBCT machines. Tube current (mA) and tube voltage (kV), field of view (FOV) size, and rotation angle were varied over the possible range. The effect of the acquisition parameters on the MGV and contrast-to-noise indicator (CNI) was analyzed by Kruskal Wallis and Dunn-Bonferroni tests for each machine independently (*α* = 0.05).

**Results:**

Tube current did not influence MGV in most machines. Viso G7 and Veraview X800 presented a decrease in the MGV for increasing kV. For ProMax 3D Max and X1, the kV did not affect the MGV. For the majority of machines, MGV decreased with increasing FOV height. In general, the rotation angle did not affect the MGV. In addition, CNI was lower with lower radiation and large FOV and did not change from 80 kV in all machines.

**Conclusions:**

The MGV and noise provided by the tested phantom vary largely among machines. The MGV is mainly influenced by the FOV size, especially for bone equivalent radiodensity. For most machines, when the acquisition parameters affect the MGV, the MGV decrease with the increase in the acquisition parameters.

## Introduction

During the reconstruction of tomographic images, each voxel receives a value according to the linear attenuation coefficient of the structure interacting with the X-ray beam. The value is obtained by averaging the pixel input values from the X-ray detector that belong to each voxel over all projection images acquired during the scan. Then, the voxel values, also named gray values (GVs), are arranged in a matrix, each representing a shade of gray in the final image. Therefore, in cone-beam computed tomography (CBCT), the GV numerically expresses the image density of structures, which does not necessarily mean the actual density.

Although it could be expected that the GV would consistently represent the actual density values of structures, such as bone, soft tissue, and air, this assumption is not true for CBCT.^1^ The high levels of radiation scatter and artifacts, limited-field geometry, and lack of standardization of CBCT machines and the acquisition parameters are issues associated with the inconsistent correlation between GV and density in CBCT. Because of these issues, the GV obtained from a CBCT machine may greatly differ from those obtained in the same or another CBCT machine.^1^^,^^2^

This inconsistency of the GV is described in the literature.^1^^,^^2^ However, how the variability of the GV occurs according to the X-ray source specifications, exposure parameters (e.g., kV and mAs), field of view (FOV), and image geometry remains largely unknown. In the literature, objective imaging analyses, such those on artifact production, are performed based on the GV. Following the methodology of Pauwels et al^3^ many authors evaluated the presence of artifacts according to exposure parameters and FOV by assessing the standard deviation (SD) of GV.^4–7^ Considering that the SD measures the variation in its mean, knowing the already expected variability of the GV could help further understand artifact production. Knowing GV behavior could also benefit other analyses performed on CBCT scans based on GV, such as radiomics, bone microarchitecture, or fractal dimension. Most importantly, this information could allow future calibration and standardization of the GV among machines. It could finally lead to the development of a more reliable scale to evaluate image density in CBCT, such as Hounsfield units (HUs) in medical CT. A recent study^8^ indicated the possibility of obtaining consistent HU values in 3 different CBCT machines through linear correlation. However, they state the need for calibration between CBCT machines, due to the differences, especially regarding different FOV sizes.

Therefore, the aim of the present study was to systematically evaluate the mean gray value (MGV) and noise provided by bone and soft tissue equivalent materials and air imaged with varied acquisition parameters in 9 CBCT machines. The null hypothesis was that the different acquisition parameters do not affect the MGV and noise.

## Methods

### Image acquisition

The DIN6868-161 (IEC 61223-3-7:2021) phantom, designed for testing the image quality of CBCT scans, was used. The phantom is composed of a polymethyl methacrylate (PMMA) cylinder of 160 mm diameter and 40 mm length, with a layer of bone equivalent structure (polyvinyl chloride of 1.30-1.45 g/cm^3^ density), a layer of soft tissue equivalent structure (PMMA of 1.20 ± 0.01 g/cm^3^), and air gap. The phantom was scanned in 9 CBCT machines: 3D Accuitomo 170 (Accuitomo) (J Morita, Kyoto, Japan), Axeos (Dentsply Sirona, Charlotte, NC, United States), Eagle 3D (Dabi Atlante, Ribeirão Preto, Brazil), NewTom 5G (NewTom) (Quantitative Radiology, Cefla, Verona, Italy), OP300 Maxio (OP300) (Instrumentarium, Tuusula, Finland), ProMax 3D Max (ProMax) (Planmeca, Helsinki, Finland), Viso G7 (Planmeca, Helsinki, Finland), X1 (3Shape, Copenhagen, Denmark), and Veraview X800 (X800) (J Morita, Kyoto, Japan). All the machines used in the study had an effective 16-bit depth of contrast resolution, and were properly calibrated, according to the local authorities’ guidelines and recommendations from the manufacturer. The voxel size was kept as the standard resolution of each machine.

The CBCT scans were acquired with varying tube current (mA), tube tension (kV), FOV size, and rotation angle, when possible (Table 1). For the machines that allowed the variation of mA (Accuitomo, Axeos, Eagle 3D, NewTom, OP300, ProMax, Viso G7, and X800), the lowest and highest mA available in the machine and an intermediate one were selected (i.e., 3 mA settings per machine), NewTom allowed only 2 variations of mA with the other parameters. kV was variable in the Accuitomo, Promax, Viso G7, X1, and X800 machines. Regarding FOV size, it was classified as small size (until 5 cm height), medium size (above 5 cm and until 10 cm height), and large size (above 10 cm height). Most machines allowed FOV variations in 3 sizes, except NewTom, OP300, X1, and X800, in which only 2 sizes were available. In NewTom was only possible to standardize the combination of 2 mA levels with 1 FOV size (i.e., 1 and 4 mA, with medium FOV) and 2 FOV sizes with 1 mA level (i.e., medium and large FOV, with 1 mA). In addition, in the X800 machine, a medium FOV size of a convex triangular FOV was selected to be compared with the medium cylindrical FOV of that machine. The rotation angle was varied at 180° and 360° in the Accuitomo and X800 machines. However, the rotation angle available for the convex triangular FOV in the X800 machine was only 180°. A CBCT scan was acquired for each protocol (i.e., each combination of the variations of the parameters allowed in the CBCT machine in every machine), totaling 246 CBCT acquisitions. Table 2 shows the specifications of the CBCT machines, according to information provided by manufacturers and previous studies.^9–12^

**Table 1.** Variations of the acquisition parameters used in each CBCT machine.

CBCT machine	Acquisition parameters				
	Tube current (mA)	Tube voltage (kV)	Field of view (cm)	Rotation angle (°)	Number of acquisitions (*N*)
3D Accuitomo 170	1, 5, 10	60, 70, 80, 90	4 × 4, 8 × 8, 14 × 10	180, 360	72
Axeos	7, 10, 13	85	5 × 5, 8 × 8, 17 × 14	315	9
Eagle 3D	4, 6.3, 8	85	5 × 5, 8 × 8, 13 × 16	360	9
NewTom 5G	1, 4	110	8 × 8, 18 × 16	360	3
OP300 Maxio	4, 6.3, 8	89.8	5 × 5, 6 × 8	360	6
ProMax 3D MAX	2, 5, 10	60, 70, 80, 90	4 × 4, 8 × 8, 23 × 16	210/360	36
Viso G7	2, 5, 9	80, 90, 100, 110, 120	7 × 4, 8 × 8, 17 × 12	210/360	45
X1	12	80, 85, 90	4 × 4, 8 × 8	360	6
Veraview X800	2, 5, 10	70, 80, 90, 100	4 × 4, 8 × 8, 10 × 8Δ	180, 360	60

Δ = Convex triangular field of view.

**Table 2.** CBCT machines’ technical specifications.

CBCT machine	Detector type	Detector position	Reconstruction Algorithm
3D Accuitomo 170	A-Si/CsI	Aligned	FBP
Axeos	A-Si/CsI	Aligned or offset^a^	FBP
Eagle 3D	CMOS/CsI	Aligned or offset^a^	FBP
NewTom 5G	A-Si/CsI	Aligned	FBP
OP300 Maxio	CMOS/CsI	Aligned	FBP
ProMax 3D MAX	A-Si/CsI	Aligned or offset^a^	FBP^c^
Viso G7	A-Si/CsI	Aligned or offset^a^	FBP^c^
X1	CMOS/CsI	Aligned	IR
Veraview X800	CMOS/CsI	Aligned or offset^b^	FBP

Detector technology: amorphous silicon (A-Si); complementary metal-oxide-semiconductor (CMOS). Scintillator material: thallium-doped caesium iodide (CsI). Reconstruction algorithm: filtered back-projection (FBP); iterative reconstruction (IR).

a Offset rotation for large field of view sizes acquisition.

b Offset rotation for convex triangular field of view acquisition.

c This machine provides an iterative reconstruction algorithm for movement-artefact correction, but it was not activated for this study.

### Image evaluation

**Figure 1.**
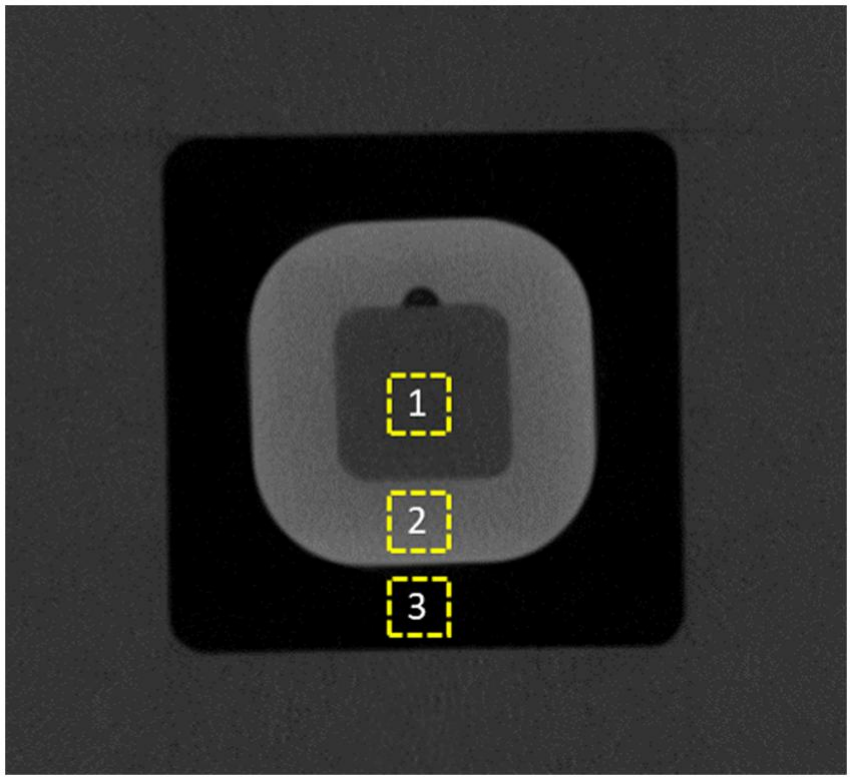
CBCT axial image of the phantom scanned in the Viso G7 machine, with 5 mA, 120 kV, and medium FOV size. Region of interest selected on the soft tissue equivalent (1), bone equivalent (2), and air (3).

With the ImageJ/Fiji software version 2.0.0 (National Institutes of Health, https://imagej.net/ij/download.html), a square region of interest (ROI) of 15 × 15 pixels was selected on the bone equivalent, soft tissue equivalent, and air layers (Figure 1). A Macro was recorded in the ImageJ/Fiji software to ensure the same location of the ROI in scans with the same FOV size from the same machine. Then, the MGV was obtained from each structure in 5 axial slices of each CBCT scan. The 5 slices were chosen from the range of slices that the phantom would entirely appear in the axial plane. Subsequently, following the methodology of Steiding et al^13^ using the same phantom, the SD of bone and soft tissue equivalent materials was also obtained to calculate the contrast-to-noise indicator (CNI). The CNI was calculated following the formula^5^^,^^13^:


$$\mathrm{CNI}=\frac{{|\text{Mean}}_{\mathrm{Bone}}-{\text{Mean}}_{\mathrm{Soft}\;\mathrm{Tissue}}|}{\sqrt{{\text{SD}}_{\mathrm{Bone}}^{2}+{\text{SD}}_{\mathrm{Soft}\;\mathrm{Tissue}}^{2}}\;}.$$

### Statistical analysis

The statistical analysis was performed using the SPSS software v. 29 (IBM SPSS Corp., Armonk, NY, United States). The Shapiro-Wilk test was applied to determine the normal distribution of the MGV and CNI. Since the data were not normally distributed, the Kruskall-Wallis test with Dunn’s *post hoc* and Bonferroni correction assessed the effect of the different conditions of mA, kV, FOV, and rotation angle on the MGV and CNI of each machine independently. The level of significance established was 5% (*α* = 0.05).

## Results

**Figure 2.**
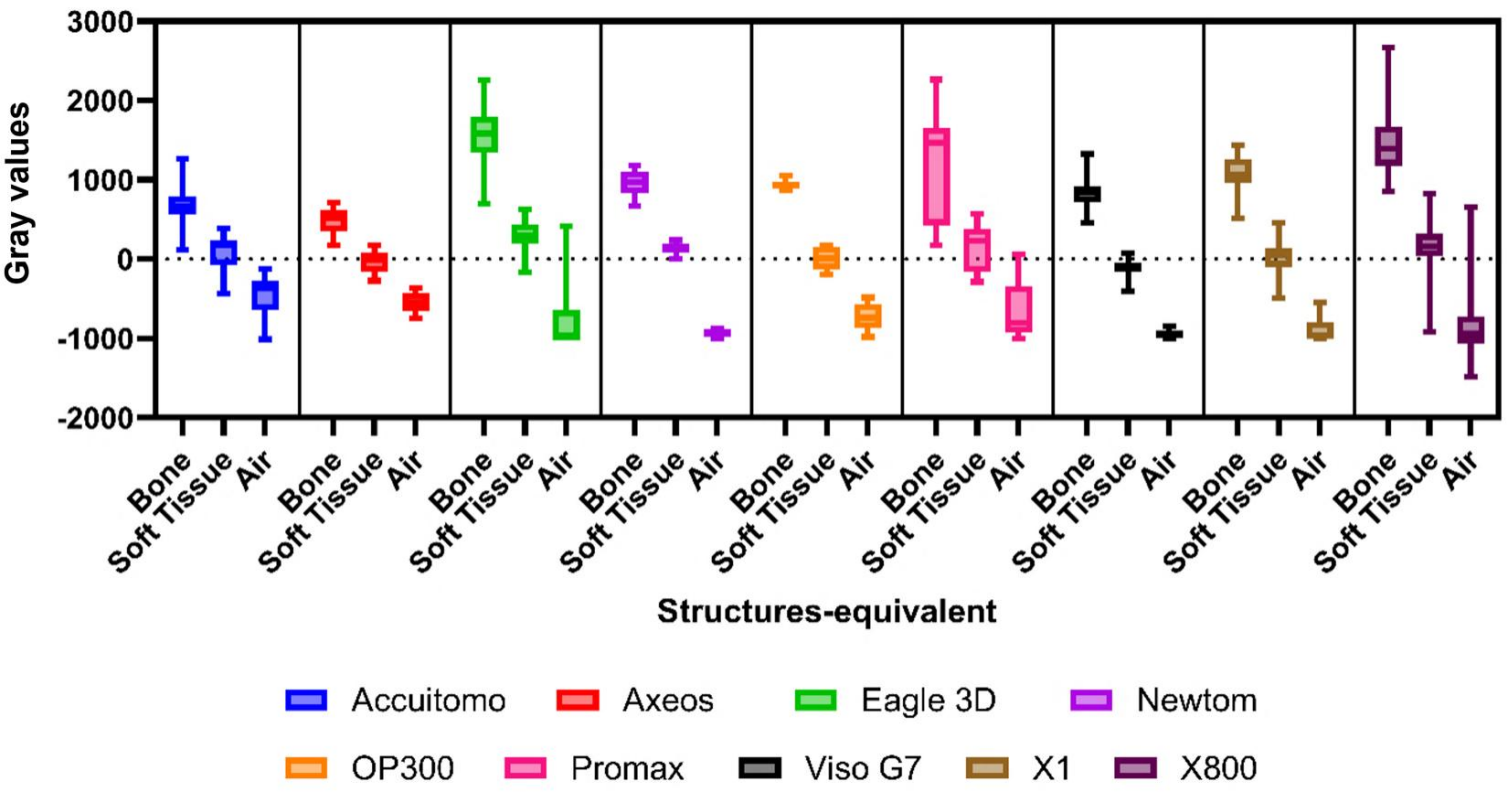
Boxplot indicating the mean gray value distribution according to the CBCT machine and structure.

Figure 2 shows the summarized MGV provided by the 3 structures according to the CBCT machine. In the boxplots, the box extends from the 25th to 75th percentiles, and the middle line in the box is plotted at the median. Lower MGV means darker or hypodense areas and higher MGV means hyperdense areas in the image evaluation. The median, minimum, and maximum MGV values according to the parameters and machines evaluated are seen in the Supplementary material.

**Figure 3.**
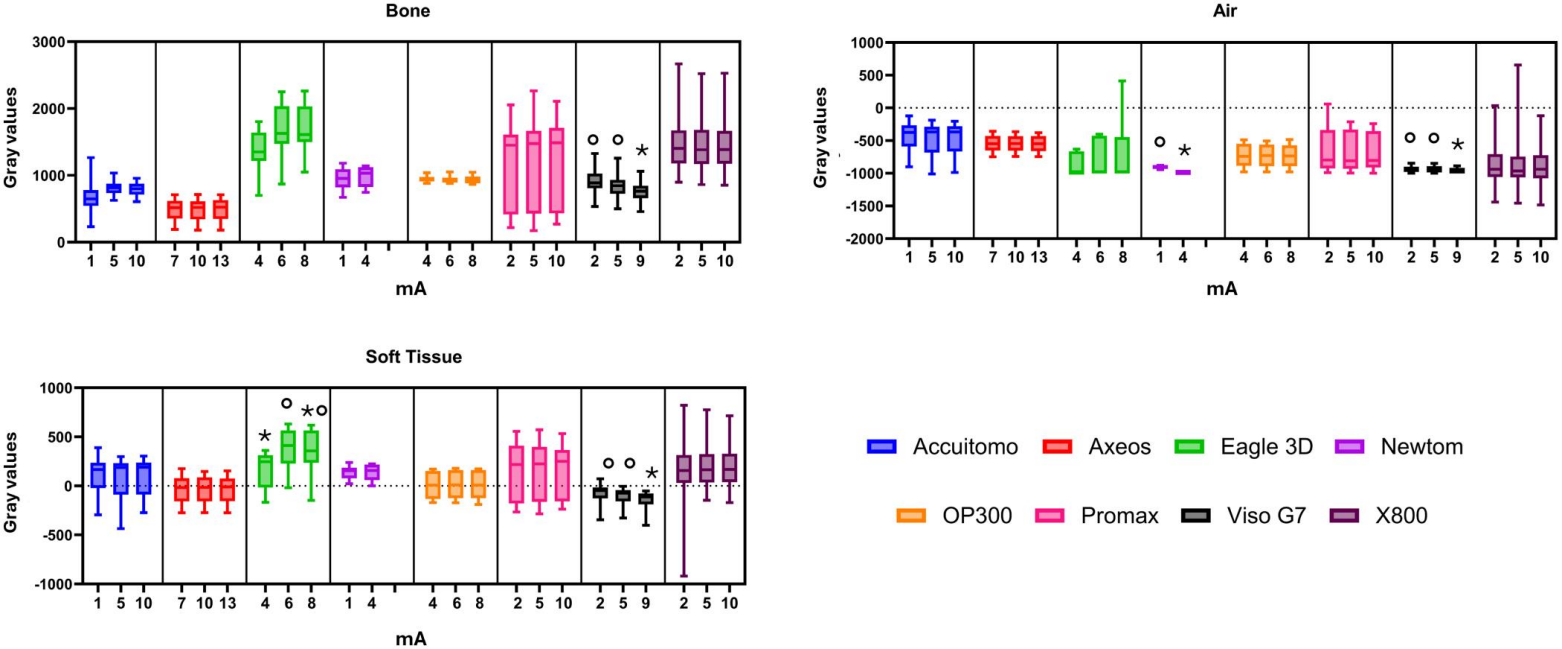
Boxplot indicating the mean gray value distribution according to the tube current (mA). Different symbols mean statistical difference (*p* ≤ 0.02).

For most CBCT machines, the mA did not affect the MGV. This parameter had similar MGV in the Accuitomo, Axeos, OP300, ProMax, and X800 machines. The Viso G7 was the only machine in which the mA affected the MGV in all 3 structures (*p* = 0.00). The high mA provided lower MGV compared to the low and intermediate mA (*p* ≤ 0.01). In addition, Eagle 3D and NewTom also showed some variance in the MGV according to the mA. For Eagle 3D, the MGV of the soft tissue of the intermediate mA was higher than the low mA (*p* = 0.02). For NewTom, the MGV for air of the low mA was higher than the intermediate mA (*p* = 0.00) (Figure 3).

**Figure 4.**
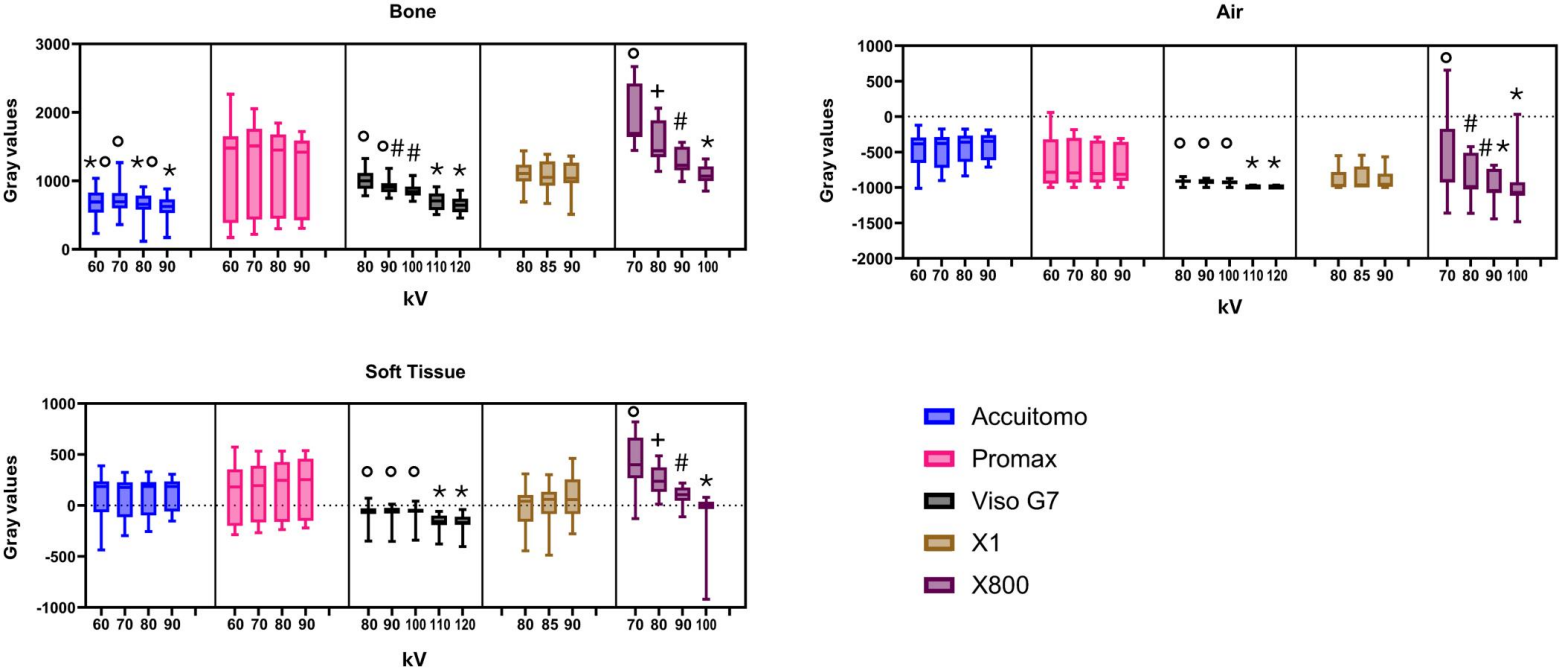
Boxplot indicating the mean gray value distribution according to the tube voltage (kV). Different symbols mean statistical difference (*p* ≤ 0.02).

Regarding the kV, for Accuitomo, 70 kV presented a higher MGV than 90 kV for bone (*p* = 0.00). For Viso G7, 110 and 120 kV had lower MGV than 80, 90, and 100 kV for all structures (*p* ≤ 0.00). Similarly, for X800, the MGV decreased when the kV increased for all structures (*p* ≤ 0.02). However, it did not influence the MGV of any structure in Promax and X1 (*p* > 0.05) (Figure 4).

**Figure 5.**
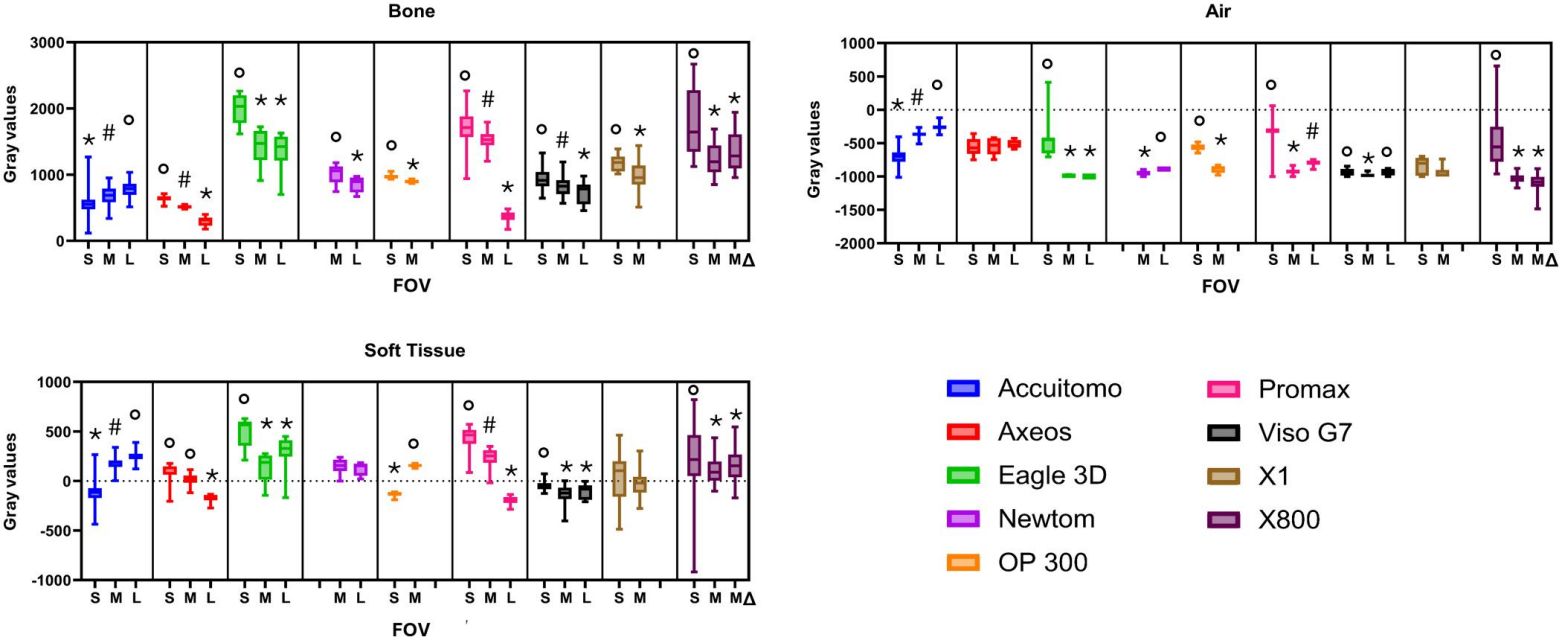
Boxplot indicating the mean gray value distribution according to the field of view (FOV) size (S = Small, M = Medium, MΔ = Medium convex triangular field of view, L = Large). Different symbols mean statistical difference (*p* ≤ 0.05).

FOV had the largest influence on MGV. Interestingly, the test material played a role in this parameter. Bone was the only structure where the MGV changed according to the FOV size in all machines. The MGV decreased when the FOV size increased (*p* ≤ 0.05) for all machines except Accuitomo, which showed the opposite behavior. Additionally, for the X800 machine, the convex triangular medium FOV did not differ from the cylindrical medium FOV for any structure (*p* > 0.08), suggesting that the FOV shape does not affect the MGV (Figure 5).

**Figure 6.**
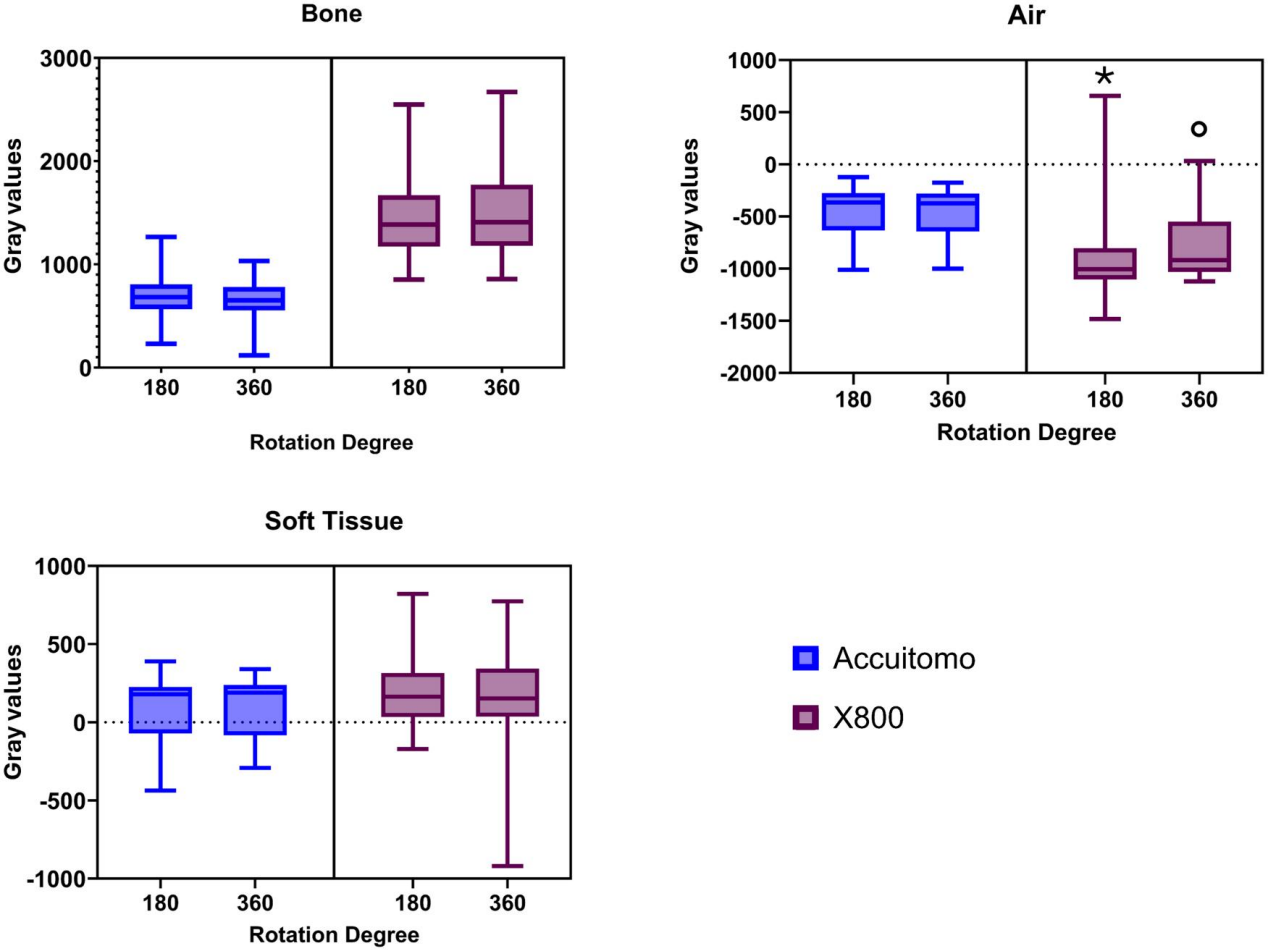
Boxplot indicating the mean gray value distribution according to the rotation angle. Different symbols mean statistical difference (*p* = 0.00).

Finally, the rotation angle of the Accuitomo and X800 machines did not affect the MGV of any structure except for air in the X800 machine (*p* = 0.00). The MGV slightly increased with 360 rotation degrees (Figure 6).

**Figure 7.**
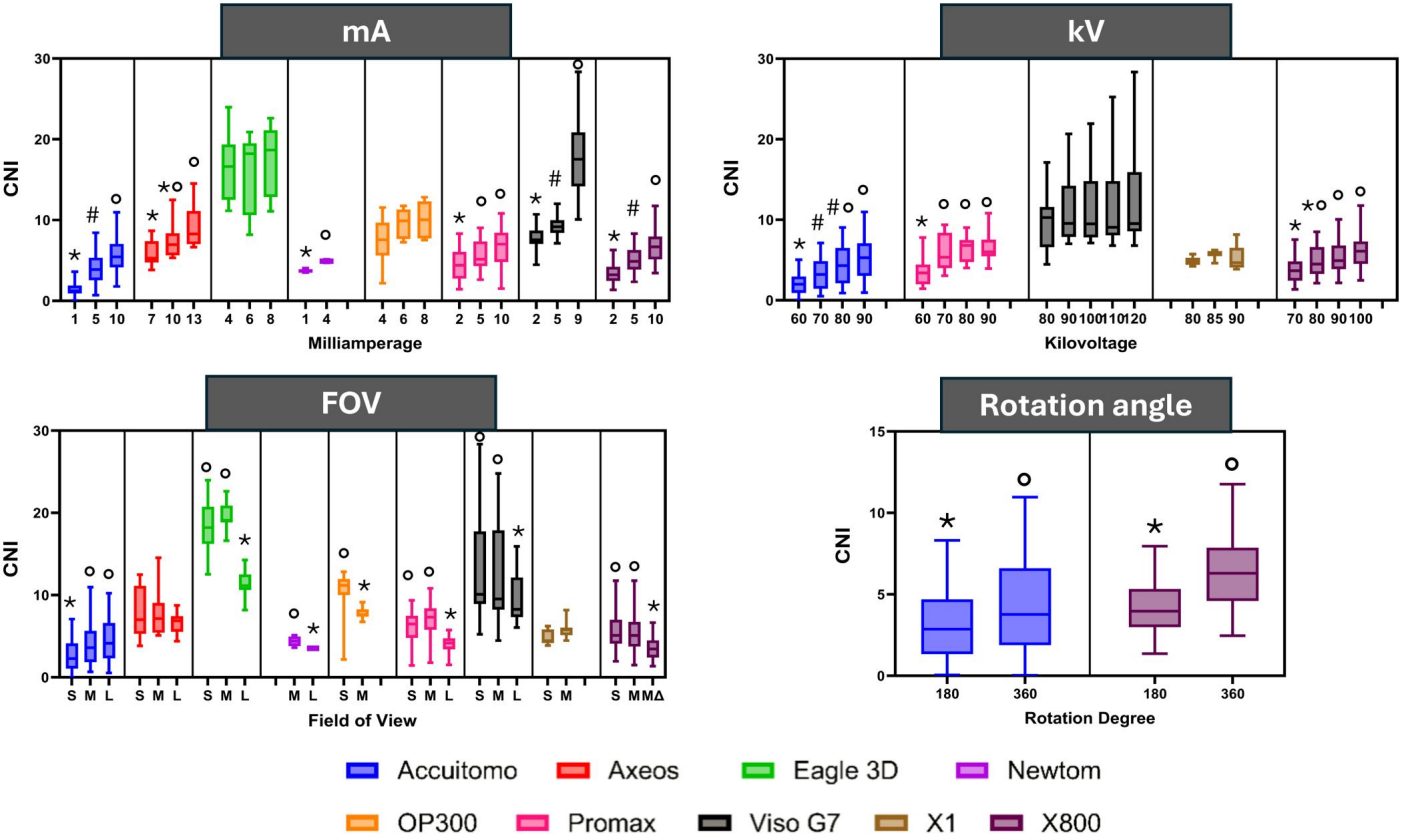
Boxplot of the CNI of each CBCT machine according to the parameters evaluated. Different symbols mean statistical difference (*p* < 0.05).

The CNI results for each machine are seen in Table 3. A higher CNI means less noise. Eagle 3D presented the highest CNI, followed by Viso G7 and OP300. In contrast, the lowest CNI was observed for Accuitomo, followed by NewTom and X800. For mA, except for Eagle 3D and OP300, the CNI increased with mA increase (*p* ≤ 0.02). The difference in kV did not affect the CNI for Viso G7 and X1(*p* > 0.11). On the other hand, for Accuitomo, ProMax, and X800, the lowest kV had the lowest CNI (*p* = 0.00). In addition, for all machines, the CNI did not change from 80 kV. Regarding the FOV, Axeos and X1 did not differ in the CNI (*p* > 0.06). All the other machines showed a lower CNI for the large FOV (*p* ≤ 0.04), except Accuitomo, in which the small FOV had a lower CNI (*p* = 0.00). Also, although the MGV was not affected by the shape of the FOV, the CNI was. The convex triangular FOV had lower CNI compared to the cylindrical FOV (*p* = 0.00). Moreover, the 180-rotation degree had lower CNI than the 360-rotation degree for both machines (*p* ≤ 0.00) (Figure 7).

**Table 3.** Contrast-to-noise indicator (CNI) median values according to acquisition parameters and CBCT machines.

		CBCT machines								
		Accuitomo	Axeos	Eagle 3D	NewTom	OP300	ProMax	Viso G7	X1	X800
Parameters	Overall	3.44	6.95	16.63	3.86	8.91	5.74	9.48	5.16	4.73
mA	Low	1.24 A	5.27 A	16.62 A	3.64 A	7.56 A	4.41 A	7.56 A	NA	3.22 A
	Intermediate	3.89 B	6.95 AB	18.21 A	4.90 B	9.92 A	5.16 B	9.17 B		4.88 B
	High	5.44 C	8.27 B	18.68 A	–	10.04 A	7.02 B	17.53 C		6.67 C
kV	60	2.00 A	NA	NA	NA	NA	3.41 A	–	–	–
	70	3.22 B					5.34 B	–	–	3.67 A
	80	4.30 BC					6.81 B	10.28 A	4.78 A	4.49 AB
	85	–					–	–	5.84 A	–
	90	5.30 C					6.00 B	9.55 A	4.68 A	4.92 B
	100	–					–	9.48 A	–	6.09 B
	110	–					–	9.07 A	–	–
	120	–					–	9.52 A	–	–
FOV	Small	2.28 A	7.02 A	18.21 B	–	11.17 B	6.48 B	10.08 B	4.47 A	5.10 B
	Medium	3.60 B	7.14 A	19.08 B	4.39 B	7.61 A	7.33 B	9.52 B	5.78 A	5.09 B
	Large^a^	4.14 B	6.88 A	11.16 A	3.53 A	–	4.16 A	8.26 A	–	3.46 A
Rotation degree	180	2.87 A	NA	NA	NA	NA	NA	NA	NA	3.97 A
	360	3.78 B								6.30 B

Different letters between the same machine and parameter represent significant differences. Letter A represents the lowest CNI median values for the same machine and parameter (*p* < 0.05).

a In the X800 machine is a medium convex triangular FOV.

## Discussion

Although the literature acknowledges the inconsistency of GV and variance among machines,^1^^,^^2^ according to the author’s knowledge, this is the first study to systematically assess the MGV and noise variance for a variety of CBCT machines. In the present study, all tested acquisition parameters affected the MGV and CNI in some machines and/or materials. Therefore, the null hypothesis was rejected. Surprisingly, even with the machine’s different reconstruction algorithms, post-processing software, and detector features, they had some similar behaviors. These similarities may be related to the filtered back-projection (FBP) algorithm used for image reconstruction in most CBCT machines available in the market.

Tube current was chosen according to the lowest, intermediate, and highest mA values available in the machine. It was not chosen based on similar mA values among the machines, once the machines were independently compared. This parameter did not affect the MGV of the machines for the 3 structures evaluated, with an exception for Viso G7. A decrease in the MGV with the higher mA was observed for this machine. Higher mA results in more X-ray photons reaching the detector. Therefore, a higher image density (i.e., darker image) would be expected, usually numerically represented by lower MGV. However, considering that this behavior was only seen for Viso G7, it suggests that this machine may have a different image processing of the raw data. The raw projection data commonly undergo various processing steps^14^ that the manufacturer does not disclose. In some manufacturers, these steps and post-processing of the final reconstructed images will largely influence the image occurrence, thus yielding unexpected effects.

Even though the different mA mostly did not affect the MGV, a decrease in the mA increased the noise. Other studies^15^^,^^16^ also observed the effect of increased noise caused by a lower radiation dose. The lower radiation dose could also explain the lower CNI for the 180° rotation angle compared to the 360° rotation angle.

When considering kV, for Viso G7, the MGV was lower for 110 and 120 kV, compared to 80, 90, and 100 kV. Similarly, for X800, the MGV decreased when the kV increased. However, for Promax and X1, the kV did not affect the MGV in any structure. Interestingly, Promax and Viso G7, and X1 and X800 have similar image detectors, which influence how the X-ray signal is read out, however different results. It is essential, then, to consider the reconstruction algorithms of the CBCT machines.

The reconstruction technique used in almost all CBCT systems is the 3D FBP by the Feldkamp-Davis-Kress algorithm^17^ due to its simplicity and fast reconstruction time.^12^^,^^14^ It consists of a ramp filter, which corrects the blur intrinsic to the projection process by filtering low frequencies and transforming them into high frequencies. Then, a smoothing filter reduces the high-frequency noise amplified by the ramp filter. Nonetheless, as a result, it ends up smoothing definitions and losing image details.^14^^,^^18^ In addition, iterative reconstruction (IR) is a new reconstruction technique that has been used in CBCT. Although IR requires more computation time and power, higher image quality can be expected by reducing image noise and artifacts.^14^^,^^18^^,^^19^ The X1 machine introduced IR algorithms as a motion-artifact correction system, not allowing acquisitions without activating this system.^10^ Therefore, the different kV results between X1 and X800 for MGV may be related to these machines’ different image reconstruction algorithms. Notwithstanding, overall the MGV of Viso G7 started to differ from 100 kV, while the kV values of Promax go until 90 kV, which means that based on the actual kV values, there was no difference between these machines.

Whether the reconstruction algorithms may be related to the MGV results, the scatter radiation may be related to the CNI results, as it is one of the factors contributing to noise production. The kV and FOV are exposure parameters associated with the scatter-primary ratio (SPR) in CBCT. Higher X-ray energies and larger FOVs increase the probability and proportion of scatter occurring, respectively.^20^ In the present study, although the differences in the CNI according to kV varied between the machines, it is possible to observe that for all machines, the CNI did not change from 80 kV, showing higher CNI compared to the lower kV levels. It suggests that the scatter radiation from 80 kV does not change enough to affect image noise. Pauwels et al^20^ did not find a difference in the SPR in relation to kV, corroborating our hypothesis.

Due to the large area detectors, CBCT scans suffer from much-scattered radiation.^21^ Detectors with complementary metal-oxide-semiconductor (CMOS) technology and terbium-activated gadolinium oxysulfide (Gadox) scintillator material will provide less image noise than amorphous silicon (A-Si) thin film transistor and thallium-doped caesium iodide (CsI), respectively. It could explain why Eagle 3D and OP300, which have a CMOS detector, were some of the machines that presented the highest CNIs. However, once again it is more important to consider the reconstruction algorithm. As mentioned above, the ramp and smoothing filter level applied in the FDK reconstruction algorithm of the CBCT machines varies according to the manufacturer’s configuration. A higher level of smoothing would decrease the image noise and, thus, increase the CNI. The authors believe that was the case for the machines with the highest CNIs. In addition, there is expected to be less image noise in the IR algorithm compared to the FDK algorithm. Although, the X1 machine was not among the highest CNIs. Nonetheless, it is important to highlight that higher CNI does not mean higher image quality.

Regarding the FOV size, it had a large influence on the MGV. It corroborates with Yadegari et al^22^ which observed a higher MGV error for a large FOV than a medium FOV. This behavior could be related to a higher scatter in larger FOVs.^20^ Oliveira et al^23^ hypothesized that the noise from scattered radiation might contribute, in part, to the inconsistency of the MGV. This hypothesis may be accurate based on the present study’s findings for the FOV size. It could also explain the lower CNI found for the large FOV.

In Accuitomo, the different behaviors observed for the MGV and the lower CNI for the small FOV might be due to the reconstruction algorithm and post-processing steps of this machine and the local tomography problem. While the MGV decreased when the FOV increased for all the other machines, we observed the opposite for the Accuitomo. Parsa et al^24^ found the same result when evaluating the MGV of Accuitomo and NewTom machines. They attributed the disparity of behaviors of the machines to the variability in reconstruction and post-processing methods applied by the manufacturers. In addition, the local tomography effect is another factor that requires attention for small FOVs.^25^ Here, the reconstructed volume is surrounded by tissue that largely attenuates the X-ray beam while not being reconstructed, called exomass.^26^ In other words, the values measured on the detector are largely influenced by tissue/material in the exomass, thus overestimating the attenuation within the FOV. This general shortcoming cannot be easily overcome, yet it will strongly influence on the MGV that are being reconstructed.

Although the FOV shape did not affect the MGV, it was not the same for the CNI. In the X800, the convex triangular FOV had a lower CNI than the cylindrical FOV. Cascante-Sequeira et al^4^ also did not find a difference in the MGV according to the FOV shape in control images (i.e., without high-density materials). However, to the best of the authors’ knowledge, the present study is the first one comparing noise for different FOV shapes. Considering that the convex triangular FOV also diminishes the radiation dose to the patient,^27^^,^^28^ it is hypothesized that the lower amount of radiation could be related to the lower CNI of the convex triangular FOV.

Interestingly, the MGV behaved differently according to the structure, especially in the FOV parameter. For the X1 machine, although the MGV decreased with the FOV size increase for all structures, only bone presented a significant difference. Besides its reconstruction algorithm, this result could also be credited to its CMOS detector, considering that this detector technology is superior to A-Si for bone evaluation, but not for soft tissue.^29^ In addition, the MGV behavior for bone structure was more standardized among the machines. That is because CBCT is mainly built for the visualization of hard-tissue contrast.

Because of the high number of machines used in the present study, their availability, and the time required for the CBCT acquisitions, only 1 exposure was performed per protocol. Considering that the machines were calibrated through image quality tests, it is believed that no significant differences would be found with a higher number of acquisitions. To collect more data, the measurements were done in 5 slices of each protocol. Therefore, the MGV of each protocol was calculated within the 5 slices. In addition, the *in vitro* nature of the present study prevents the direct extrapolation of the results to a clinical scenario. Although the phantom used consisted of materials with similar MGV of patients’ bone and soft tissue, it is challenging to completely mimic the X-ray attenuation of a patient’s head. However, the *in vitro* approach allowed the control of the assessed variables and a standardized object of evaluation. Moreover, the present results provide valuable insights into the MGV behavior among the CBCT machines. Further studies are encouraged to explore this behavior in a clinical scenario, with artifact production, activation of metal artifact reduction tools, and new machines. Notwithstanding, the present study indicates the expected GV behavior in different exposure conditions, having this knowledge hold potential for future calibration of MGV among CBCT machines.

## Conclusion

The mean gray values and noise provided by the tested phantom vary largely among machines. Gray values are mainly influenced by the FOV size, especially for bone equivalent radiodensity. Mostly, noise increases with lower radiation, it does not change from 80 kV, and it is larger in a large FOV. For most machines, when the exposure parameters or FOV affect the gray values, the gray values decrease with the increase in the exposure parameters and/or FOV height.

## Supplementary Material

twae053_Supplementary_Data
