# Supplementary material for: Gray values and noise behavior of cone-beam computed tomography machines—an in vitro study
Source: Dentomaxillofac Radiol. 2024 Nov 19;54(2):140–8. doi: 10.1093/dmfr/twae053 (PMC11784915; doi:10.1093/dmfr/twae053)
Supplement: twae053_Supplementary_Data [file twae053_supplementary_data.docx]

**Supplementary material** Median (minimum-maximum) gray values according to the acquisition parameters in the machines evaluated
